# Supplementary material for: In Vivo Anti-Inflammatory Effects and Related Mechanisms of Processed Egg Yolk, a Potential Anti-Inflammaging Dietary Supplement
Source: Nutrients. 2020 Sep 4;12(9):2699. doi: 10.3390/nu12092699 (PMC7551027; doi:10.3390/nu12092699)
Supplement: Supplementary file 1 [file nutrients-12-02699-s001.zip › SUPPLEMENTAL FIGURE 1.docx]

SUPPLEMENTAL FIGURE 1


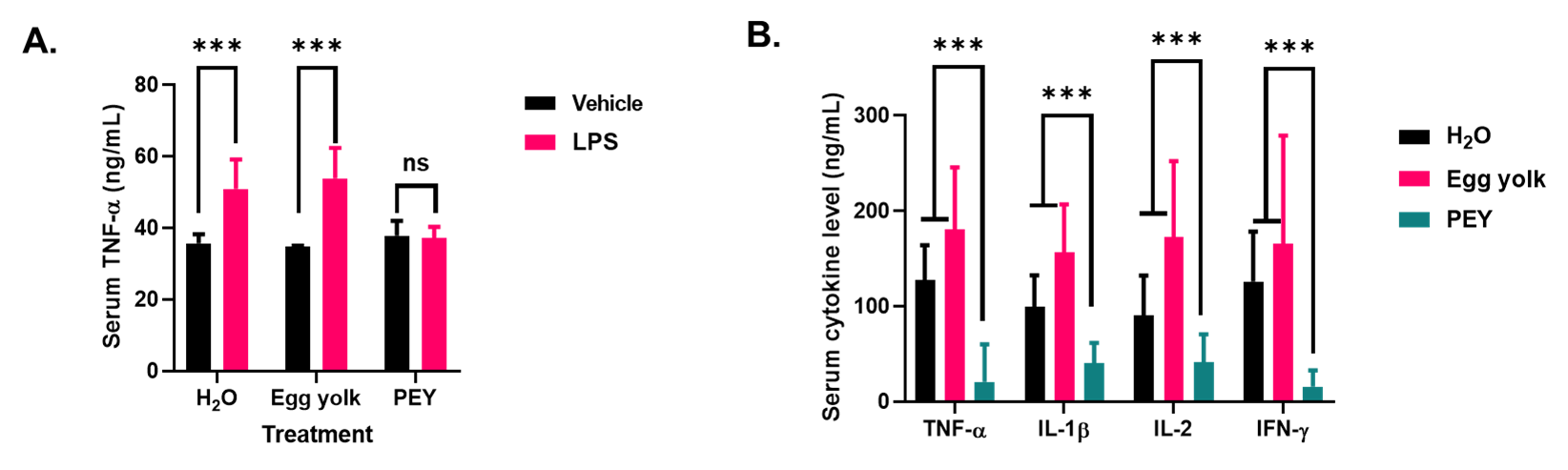


**Supplemental Figure 1.** A). Oral administration of PEY during one day is able to prevent LPS-induced TNF-α increase in serum in comparison with water or egg yolk. B) Oral administration during two days is able to prevent LPS-induced increase of serum cytokines IL-1β, TNF-α, IFN-γ, and IL-2. *** indicates significant differences between groups (p<0.001) in two- way ANOVA post-hoc analyses after Benjamini, Krieger and Yekutelli correction for false discovery rate. In all cases, 5 animals were treated per group.
